# The Hospital World

**Published:** 1910-07-30

**Authors:** 


### The Hospital World.

### Elections and Appointments.

Mr. Cornelius Buckley has been appointed accountant at the North Infirmary, Cork.

Canon H. T. Ottley has been elected a member of the committee of Bromyard Cottage Hospital.

Mr. A. Moore has been appointed secretary and superintendent of the Lincoln County Hospital.

The Earl of Plymouth has been elected president of the Birmingham and Midland Eye Hospital.

Mr. C. H. Wright has been re-appointed auditor to the Sister Dora Convalescent Hospital, Milford.

Mr. Alderman Wherry, J.P., of Bourne, Lincolnshire, has been appointed a life governor of Leicester Infirmary.

Mr. D. Wilberforce-Smith, M.B., B.S., has been appointed assistant registrar to the Samaritan Free Hospital, London.

Mr. Alderman George Hirst and Mr. Councillor W. A. Parker have been appointed members of the Dewsbury Joint Hospital Board.

The following gentlemen have been appointed governors of the Bainsley Beckett Hospital, Sheffield: Mr. G. A. Bond, the Rev. T. T. Taylor, the Rev. W. R. Hervey, Mr. H. Carter, Mr. F. Brown, and Mr. J. Elliott, together with the following representatives of the Saturday and Sunday Committee: Mr. A. Whitham, Mr. J. S. Rose, Mr. H. Feasby, Mr. C. B. Moore (Stainborough), Mr. J. Cope (Pogmoor), and Mr. C. Page (Darfield).

The following ladies and gentlemen have been elected on the Board of Management of the Swansea Hospital for the ensuing twelve months: Dr. Ebenezer Davies, Mr. T. Howell Davies, Mr. Evan Evans, Rev. R. B. Gwydr, Mr. Hyam Goldberg, Mrs. David Harris, Mr. Geo. Jenkins, Mr. R. W. Jones, Mr. Richard Lewis, Mr. Henry Macdonnell, Mrs. R. Martin, Mr. David Meager, the Rev. H. C. Mander, Mr. Sidney Palmer, Mrs. C. H. Perkins, the Rev. John Pollock, Mr. J. Brook Pritchard, Mr. C. H. Quick, Mrs. E. E. Roberts, Mr. David Salmon, the Rev. H. J. Sandheim, Mr. H. G. Thomas, Mr. E. Montgomery Williams, and Mrs. E. M. Williams.

### Personalia.

The death of Mr. Thomas Sutton Timmis removes a philanthropist to whom not Liverpool only, but the North of England also, owes a debt. The Liverpool Royal Infirmary, of which he had been president, made him a life trustee, and received from him constant sums of money, among which we may mention the £500 he gave towards the building fund and the £1,000 towards the new outpatient department. Mr. Timmis was, moreover, one of the early rich persons to endow cancer research, which he did with the great sum of £10,000, by presenting this amount to the university in 1903. He gained his wealth as a manufacturer of soap, and vied with his partner, Mr. Gossage, in his generosity to the town and neighbourhood of Liverpool. The quantitative laboratory at Liverpool University was a present from the two, and many other departments of life benefited at their hands. It is with Mr. Timmis's work for hospitals, however, that we are concerned here. Few can give large sums of money to the hospitals, and these few must remember that Mr. Timmis, for all his generosity, did not scorn the burden and heat of routine work, and this note has pointed out two offices in a hospital which he personally held, and for his work in which the Liverpool Royal Infirmary will remember him gratefully.

A remarkable instance of continuous family service is furnished from Guy's Hospital. Mr. A. Jackson Prestage has retired from the works department after about fifty years' service. His reminiscences should be worth recording, and we commend him to the notice of the institutional *Gazette*. Mr. Prestage's father worked at the hospital for fifty years, and his grandfather and great-grandfather considerably longer.

By way of commemorating his four years' presidency of the Dewsbury Dispensary, Major Walker has promised to pay the difference between the original cost of the extension scheme and the cost of a modified scheme which want of money had forced upon the board. Major Walker's gift will amount to about £120, and he and the Dispensary are to be congratulated on his generosity, which, by taking this practical form, shows a thorough appreciation of the office of president, which will remain an example to all who succeed.

